# Bis(2,1,3-benzoselenadiazole-κ*N*)dichloridozinc(II)

**DOI:** 10.1107/S1600536808026366

**Published:** 2008-08-20

**Authors:** Hoong-Kun Fun, Annada C. Maity, Sibaprasad Maity, Shyamaprosad Goswami, Suchada Chantrapromma

**Affiliations:** a X-ray Crystallography Unit, School of Physics, Universiti Sains Malaysia, 11800 USM, Penang, Malaysia; b Department of Chemistry, Bengal Engineering and Science University, Shibpur, Howrah 711 103, India; c Crystal Materials Research Unit, Department of Chemistry, Faculty of Science, Prince of Songkla University, Hat-Yai, Songkhla 90112, Thailand

## Abstract

In the title complex, [ZnCl_2_(C_6_H_4_N_2_Se)_2_], the Zn^II^ center is tetra­coordinated by a Cl_2_N_2_ donor set in a distorted tetrahedral geometry. Some of the distortion from the ideal tetrahedral geometry might be ascribed to two agostic Z⋯H interactions The two 2,1,3-benzoselenadiazole ligands are each essentially planar and form a dihedral angle of 35.06 (9)°.   An interesting feature of the crystal packing is the observation of short intermolecular contacts between Se and Se, Se and N, and N and N atoms. These arise as a result of three-center bridging of adjacent molecules into chains along the *b* axis. The crystal structure is stablilized by π–π inter­actions [minimum centroid–centroid distance = 3.5694 (18) Å].

## Related literature

For related literature and applications of the 2,1,3-benzo­selenadiazole mol­ecule and its metal complexes, see, for example: Galet *et al.* (1994 ▶); Grivas (2000 ▶); Iwaoka & Tomoda (1994 ▶, 2000 ▶); Saiki *et al.* (1997 ▶); Zhou *et al.* (2005 ▶).
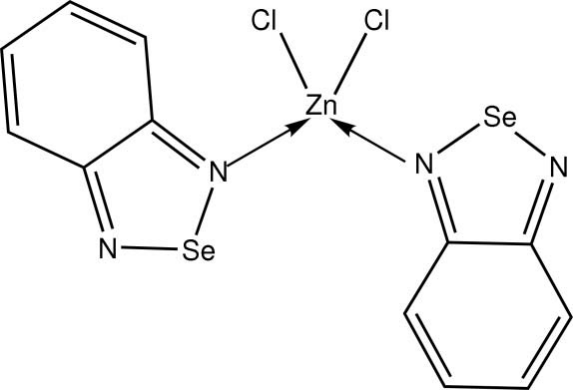


## Experimental

### 

#### Crystal data

[ZnCl_2_(C_6_H_4_N_2_Se)_2_]
*M* *_r_* = 502.43
Triclinic, 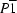

*a* = 7.5593 (2) Å
*b* = 9.7269 (3) Å
*c* = 10.6083 (3) Å
α = 95.103 (1)°
β = 92.581 (1)°
γ = 101.713 (1)°
*V* = 759.15 (4) Å^3^
*Z* = 2
Mo *K*α radiation
μ = 6.76 mm^−1^
*T* = 297 (2) K
0.48 × 0.32 × 0.30 mm

#### Data collection

Bruker SMART APEXII CCD area-detector diffractometer
Absorption correction: multi-scan (*SADABS*; Bruker, 2005 ▶) *T*_min_ = 0.141, *T*_max_ = 0.236 (expected range = 0.079–0.132)
17787 measured reflections
4425 independent reflections
3836 reflections with *I* > 2σ(*I*)
*R*_int_ = 0.035

#### Refinement

*R*[*F* ^2^ > 2σ(*F* ^2^)] = 0.032
*wR*(*F* ^2^) = 0.094
*S* = 1.06
4425 reflections
191 parameters
H-atom parameters constrained
Δρ_max_ = 0.79 e Å^−3^
Δρ_min_ = −0.54 e Å^−3^

### 

Data collection: *APEX2* (Bruker, 2005 ▶); cell refinement: *APEX2*; data reduction: *SAINT* (Bruker, 2005 ▶); program(s) used to solve structure: *SHELXTL* (Sheldrick, 2008 ▶); program(s) used to refine structure: *SHELXTL*; molecular graphics: *SHELXTL*; software used to prepare material for publication: *SHELXTL* and *PLATON* (Spek, 2003 ▶).

## Supplementary Material

Crystal structure: contains datablocks global, I. DOI: 10.1107/S1600536808026366/tk2296sup1.cif

Structure factors: contains datablocks I. DOI: 10.1107/S1600536808026366/tk2296Isup2.hkl

Additional supplementary materials:  crystallographic information; 3D view; checkCIF report

## Figures and Tables

**Table 1:** Selected interatomic distances (Å)

Se1⋯Se2^i^	3.7002 (4)
Se1⋯N4^i^	2.893 (2)
Se2⋯N2^ii^	2.918 (2)
N2⋯N4^i^	2.882 (3)
Se1⋯Cl1	3.4111 (8)
Se2⋯Cl2	3.4192 (9)
Cl1⋯N1	3.293 (2)
Zn1⋯H2*B*	3.23
Zn1⋯H8B	3.26

Symmetry codes: (i) 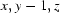; (ii) 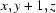.

**Fig. 1.**
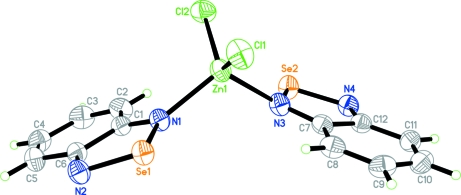
The molecular structure of (I), showing 50% probability displacement ellipsoids and the atomic numbering.

**Fig. 2.**
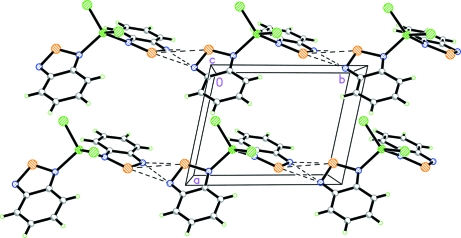
The crystal packing of (I), viewed along the c axis showing chains running along the [0 1 0] direction. Intermolecular contacts are shown as dashed lines (see Comment).

## supplementary crystallographic information

### Comment

Metal complexes of 2,1,3-benzoselenadiazole continue to attract the interest of inorganic chemists (e.g. Grivas, 2000). Organoselenium derivatives stabilized by non-bonded Se···N interactions (Galet *et al.*, 1994); Iwaoka & Tomoda, 1994; 2000; Saiki *et al.*, 1997) are also of interest because of their vital roles in many chemical phenomena, such as molecular recognition and molecular packing in crystal phases as well as due to their biological roles (Zhou *et al.*, 2005). The reaction of 2,1,3-benzoselenadiazole with ZnCl_2_ results in the formation of the title zinc(II) complex (I).

The structure of (I) comprises a neutral ZnCl_2_*L*_2_ molecule (*L*= 2,1,3-benzoselenadiazole ligand), Fig. 1. The Zn^II^ ion is tetra-coordinated by two Cl^-^ ions and two N atoms derived from the L ligands. The L ligands are each essentially planar with the maximum deviation of 0.028 (2)Å being for atom N1 in one ligand and 0.044 (2) Å for the N3 atom in the other ligand. The dihedral angle between the their mean planes is 35.06 (9)°. The distorted tetrahedral geometry can be indicated by the bond angles subtended at Zn: N—Zn—N = 111.13 (9)°, Cl—Zn—Cl = 122.58 (4)°, and N—Zn—Cl in the range of 100.70 (6) - 110.81 (7)°. Some of the distortion from the ideal tetrahedral geometry might be ascribed to two agostic Zn···H interactions, Table 1.

The interesting feature of the crystal packing is the observation of short intermolecular contacts between Se and Se, Se and N, and N and N atoms (Table 1). These arise as a result of three-center bridging of adjacent molecules into chains along the *b*-axis, Fig. 2. The crystal is further stabilized by π–π interactions with the shortest of these being 3.5694 (18) Å for Cg(C7/C12/N4/Se2/N3)···*Cg*(C7–C12)^i^ for i: -*x*, 2-*y*, -*z*.

### Experimental

A slurry of 2,1,3-benzoselenadiazole (1 g, 5.4 mmol) and anhydrous zinc chloride (270 mg, 2.72 mmol) in dry methanol (15 ml) was heated at 343–353 K for 2 h. After completion of the reaction, the mixture was allowed to cool to room temperature and the precipitate (I) was collected by filtration. Recrystallization of (I) from 40% methanol in chloroform afforded a yellow microcrystalline solid (1.16 g, 85% yield).

### Refinement

All H atoms were placed in calculated positions with C—H = 0.93 Å, and with *U*_iso_=1.2*U*_eq_(C).

### Crystal data

**Table:**

[ZnCl_2_(C_6_H_4_N_2_Se)_2_]	*Z* = 2
*M**_r_* = 502.43	*F*_000_ = 480
Triclinic, *P*1	*D*_x_ = 2.198 Mg m^−^^3^
Hall symbol: -P 1	Mo *K*α radiation λ = 0.71073 Å
*a* = 7.5593 (2) Å	Cell parameters from 4425 reflections
*b* = 9.7269 (3) Å	θ = 1.9–30.0º
*c* = 10.6083 (3) Å	µ = 6.76 mm^−^^1^
α = 95.103 (1)º	*T* = 297 (2) K
β = 92.581 (1)º	Block, yellow
γ = 101.713 (1)º	0.48 × 0.32 × 0.30 mm
*V* = 759.15 (4) Å^3^	

### Data collection

**Table:**

Bruker SMART APEXII CCD area-detector diffractometer	4425 independent reflections
Radiation source: fine-focus sealed tube	3836 reflections with *I* > 2σ(*I*)
Monochromator: graphite	*R*_int_ = 0.035
Detector resolution: 8.33 pixels mm^-1^	θ_max_ = 30.0º
*T* = 297(2) K	θ_min_ = 1.9º
ω scans	*h* = −10→10
Absorption correction: multi-scan(SADABS; Bruker, 2005)	*k* = −13→13
*T*_min_ = 0.141, *T*_max_ = 0.236	*l* = −14→14
17787 measured reflections	

### Refinement

**Table:**

Refinement on *F*^2^	Hydrogen site location: inferred from neighbouring sites
Least-squares matrix: full	H-atom parameters constrained
*R*[*F*^2^ > 2σ(*F*^2^)] = 0.032	*w* = 1/[σ^2^(*F*_o_^2^) + (0.0556*P*)^2^ + 0.3045*P*] where *P* = (*F*_o_^2^ + 2*F*_c_^2^)/3
*wR*(*F*^2^) = 0.094	(Δ/σ)_max_ = 0.001
*S* = 1.06	Δρ_max_ = 0.79 e Å^−^^3^
4425 reflections	Δρ_min_ = −0.54 e Å^−^^3^
191 parameters	Extinction correction: SHELXTL (Sheldrick, 2008), Fc^*^=kFc[1+0.001xFc^2^λ^3^/sin(2θ)]^-1/4^
Primary atom site location: structure-invariant direct methods	Extinction coefficient: 0.0238 (15)
Secondary atom site location: difference Fourier map	

### Special details

**Table:**

Geometry. All e.s.d.'s (except the e.s.d. in the dihedral angle between two l.s. planes) are estimated using the full covariance matrix. The cell e.s.d.'s are taken into account individually in the estimation of e.s.d.'s in distances, angles and torsion angles; correlations between e.s.d.'s in cell parameters are only used when they are defined by crystal symmetry. An approximate (isotropic) treatment of cell e.s.d.'s is used for estimating e.s.d.'s involving l.s. planes.
Refinement. Refinement of *F*^2^ against ALL reflections. The weighted *R*-factor *wR* and goodness of fit *S* are based on *F*^2^, conventional *R*-factors *R* are based on *F*, with *F* set to zero for negative *F*^2^. The threshold expression of *F*^2^ > σ(*F*^2^) is used only for calculating *R*-factors(gt) *etc*. and is not relevant to the choice of reflections for refinement. *R*-factors based on *F*^2^ are statistically about twice as large as those based on *F*, and *R*- factors based on ALL data will be even larger.

### Fractional atomic coordinates and isotropic or equivalent isotropic displacement parameters (Å^2^)

**Table:**

	*x*	*y*	*z*	*U*_iso_*/*U*_eq_	
Zn1	0.28675 (5)	0.75148 (3)	0.25160 (3)	0.04191 (10)	
Se1	0.12303 (4)	0.41702 (3)	0.29459 (2)	0.04073 (9)	
Se2	0.15677 (4)	1.04989 (3)	0.33867 (3)	0.04686 (10)	
Cl1	0.27521 (13)	0.62664 (8)	0.06430 (7)	0.05663 (19)	
Cl2	0.53270 (11)	0.90507 (8)	0.33168 (8)	0.05477 (18)	
N1	0.2184 (3)	0.5929 (2)	0.3668 (2)	0.0382 (4)	
N2	0.1617 (3)	0.3407 (2)	0.4366 (2)	0.0440 (5)	
N3	0.0973 (3)	0.8770 (2)	0.2511 (2)	0.0423 (5)	
N4	−0.0058 (3)	1.1149 (2)	0.2476 (2)	0.0439 (5)	
C1	0.2687 (3)	0.5849 (3)	0.4871 (2)	0.0369 (5)	
C2	0.3457 (4)	0.7016 (3)	0.5778 (3)	0.0484 (6)	
H2B	0.3642	0.7937	0.5561	0.058*	
C3	0.3908 (5)	0.6740 (4)	0.6958 (3)	0.0549 (7)	
H3A	0.4396	0.7494	0.7558	0.066*	
C4	0.3669 (4)	0.5338 (4)	0.7330 (3)	0.0529 (7)	
H4B	0.4025	0.5203	0.8153	0.063*	
C5	0.2933 (4)	0.4204 (3)	0.6502 (3)	0.0495 (6)	
H5A	0.2780	0.3295	0.6747	0.059*	
C6	0.2399 (4)	0.4432 (3)	0.5250 (2)	0.0385 (5)	
C7	−0.0380 (4)	0.8768 (3)	0.1657 (2)	0.0391 (5)	
C8	−0.1276 (4)	0.7592 (3)	0.0802 (3)	0.0453 (6)	
H8A	−0.0962	0.6717	0.0811	0.054*	
C9	−0.2595 (4)	0.7790 (3)	−0.0020 (3)	0.0495 (6)	
H9A	−0.3217	0.7025	−0.0564	0.059*	
C10	−0.3070 (4)	0.9128 (4)	−0.0086 (3)	0.0513 (7)	
H10A	−0.3962	0.9217	−0.0687	0.062*	
C11	−0.2262 (4)	1.0266 (3)	0.0700 (3)	0.0448 (6)	
H11A	−0.2582	1.1133	0.0639	0.054*	
C12	−0.0907 (4)	1.0119 (3)	0.1627 (2)	0.0393 (5)	

### Atomic displacement parameters (Å^2^)

**Table:**

	*U*^11^	*U*^22^	*U*^33^	*U*^12^	*U*^13^	*U*^23^
Zn1	0.05150 (19)	0.03211 (16)	0.04206 (17)	0.00843 (12)	0.00312 (13)	0.00397 (11)
Se1	0.04956 (16)	0.03208 (14)	0.03864 (14)	0.00715 (10)	−0.00216 (10)	−0.00116 (9)
Se2	0.05777 (18)	0.03321 (15)	0.04748 (16)	0.00941 (12)	−0.00718 (12)	−0.00210 (10)
Cl1	0.0774 (5)	0.0494 (4)	0.0440 (3)	0.0175 (4)	0.0075 (3)	−0.0020 (3)
Cl2	0.0527 (4)	0.0431 (4)	0.0637 (4)	0.0004 (3)	0.0045 (3)	0.0012 (3)
N1	0.0460 (11)	0.0290 (9)	0.0388 (10)	0.0070 (8)	0.0016 (8)	0.0008 (8)
N2	0.0537 (13)	0.0324 (10)	0.0454 (11)	0.0082 (9)	0.0022 (10)	0.0035 (8)
N3	0.0533 (13)	0.0281 (9)	0.0442 (11)	0.0069 (9)	0.0002 (9)	0.0010 (8)
N4	0.0498 (12)	0.0323 (10)	0.0494 (12)	0.0090 (9)	0.0025 (10)	0.0020 (9)
C1	0.0375 (11)	0.0341 (11)	0.0383 (11)	0.0067 (9)	0.0021 (9)	0.0014 (9)
C2	0.0534 (15)	0.0393 (13)	0.0474 (14)	0.0017 (11)	0.0018 (12)	−0.0048 (11)
C3	0.0552 (17)	0.0569 (18)	0.0453 (14)	0.0023 (14)	−0.0042 (13)	−0.0100 (13)
C4	0.0503 (16)	0.0693 (19)	0.0377 (13)	0.0110 (14)	−0.0035 (11)	0.0038 (12)
C5	0.0568 (16)	0.0506 (15)	0.0433 (13)	0.0138 (13)	0.0006 (12)	0.0110 (12)
C6	0.0402 (12)	0.0366 (12)	0.0390 (11)	0.0084 (9)	0.0028 (9)	0.0043 (9)
C7	0.0423 (12)	0.0319 (11)	0.0413 (12)	0.0034 (9)	0.0053 (10)	0.0032 (9)
C8	0.0503 (15)	0.0334 (12)	0.0494 (14)	0.0038 (11)	0.0075 (12)	−0.0019 (10)
C9	0.0450 (14)	0.0497 (15)	0.0477 (14)	0.0010 (12)	0.0041 (11)	−0.0080 (12)
C10	0.0436 (14)	0.0647 (19)	0.0455 (14)	0.0127 (13)	0.0015 (11)	0.0025 (13)
C11	0.0437 (13)	0.0465 (14)	0.0464 (13)	0.0132 (11)	0.0058 (11)	0.0071 (11)
C12	0.0416 (12)	0.0340 (11)	0.0421 (12)	0.0066 (9)	0.0070 (10)	0.0032 (9)

### Geometric parameters (Å, °)

**Table:**

Zn1—N1	2.052 (2)	C3—C4	1.433 (5)
Zn1—N3	2.062 (2)	C3—H3A	0.9300
Zn1—Cl2	2.2169 (8)	C4—C5	1.353 (5)
Zn1—Cl1	2.2231 (8)	C4—H4B	0.9300
Se1—N2	1.776 (2)	C5—C6	1.420 (4)
Se1—N1	1.803 (2)	C5—H5A	0.9300
Se2—N4	1.777 (2)	C7—C8	1.427 (4)
Se2—N3	1.809 (2)	C7—C12	1.451 (3)
N1—C1	1.328 (3)	C8—C9	1.349 (4)
N2—C6	1.330 (3)	C8—H8A	0.9300
N3—C7	1.335 (4)	C9—C10	1.425 (4)
N4—C12	1.324 (4)	C9—H9A	0.9300
C1—C2	1.428 (4)	C10—C11	1.344 (4)
C1—C6	1.447 (3)	C10—H10A	0.9300
C2—C3	1.347 (5)	C11—C12	1.425 (4)
C2—H2B	0.9300	C11—H11A	0.9300
			
Se1···Se2^i^	3.7002 (4)	Se2···Cl2	3.4192 (9)
Se1···N4^i^	2.893 (2)	Cl1···N1	3.293 (2)
Se2···N2^ii^	2.918 (2)	Zn1···H2B	3.23
N2···N4^i^	2.882 (3)	Zn1···H8B	3.26
Se1···Cl1	3.4111 (8)		
			
N1—Zn1—N3	111.13 (9)	C5—C4—H4B	119.5
N1—Zn1—Cl2	110.81 (7)	C3—C4—H4B	119.5
N3—Zn1—Cl2	101.44 (7)	C4—C5—C6	118.6 (3)
N1—Zn1—Cl1	100.70 (6)	C4—C5—H5A	120.7
N3—Zn1—Cl1	110.32 (7)	C6—C5—H5A	120.7
Cl2—Zn1—Cl1	122.58 (4)	N2—C6—C5	124.0 (2)
N2—Se1—N1	92.42 (10)	N2—C6—C1	115.9 (2)
N4—Se2—N3	92.48 (11)	C5—C6—C1	120.1 (2)
C1—N1—Se1	108.40 (16)	N3—C7—C8	125.9 (2)
C1—N1—Zn1	130.93 (18)	N3—C7—C12	114.2 (2)
Se1—N1—Zn1	118.64 (11)	C8—C7—C12	119.9 (2)
C6—N2—Se1	108.54 (17)	C9—C8—C7	117.9 (3)
C7—N3—Se2	108.30 (17)	C9—C8—H8A	121.1
C7—N3—Zn1	129.73 (18)	C7—C8—H8A	121.1
Se2—N3—Zn1	117.85 (13)	C8—C9—C10	122.4 (3)
C12—N4—Se2	108.45 (18)	C8—C9—H9A	118.8
N1—C1—C2	125.9 (2)	C10—C9—H9A	118.8
N1—C1—C6	114.7 (2)	C11—C10—C9	121.8 (3)
C2—C1—C6	119.4 (2)	C11—C10—H10A	119.1
C3—C2—C1	118.0 (3)	C9—C10—H10A	119.1
C3—C2—H2B	121.0	C10—C11—C12	118.8 (3)
C1—C2—H2B	121.0	C10—C11—H11A	120.6
C2—C3—C4	122.9 (3)	C12—C11—H11A	120.6
C2—C3—H3A	118.6	N4—C12—C11	124.3 (2)
C4—C3—H3A	118.6	N4—C12—C7	116.5 (2)
C5—C4—C3	121.0 (3)	C11—C12—C7	119.2 (2)
			
N2—Se1—N1—C1	−0.83 (19)	C3—C4—C5—C6	0.0 (5)
N2—Se1—N1—Zn1	164.72 (14)	Se1—N2—C6—C5	−178.8 (2)
N3—Zn1—N1—C1	−95.1 (2)	Se1—N2—C6—C1	1.4 (3)
Cl2—Zn1—N1—C1	16.9 (2)	C4—C5—C6—N2	−178.1 (3)
Cl1—Zn1—N1—C1	148.1 (2)	C4—C5—C6—C1	1.6 (4)
N3—Zn1—N1—Se1	103.20 (13)	N1—C1—C6—N2	−2.2 (3)
Cl2—Zn1—N1—Se1	−144.82 (10)	C2—C1—C6—N2	177.7 (2)
Cl1—Zn1—N1—Se1	−13.67 (13)	N1—C1—C6—C5	178.0 (2)
N1—Se1—N2—C6	−0.4 (2)	C2—C1—C6—C5	−2.0 (4)
N4—Se2—N3—C7	−1.8 (2)	Se2—N3—C7—C8	−178.1 (2)
N4—Se2—N3—Zn1	157.59 (14)	Zn1—N3—C7—C8	25.8 (4)
N1—Zn1—N3—C7	−100.4 (2)	Se2—N3—C7—C12	2.9 (3)
Cl2—Zn1—N3—C7	141.8 (2)	Zn1—N3—C7—C12	−153.21 (19)
Cl1—Zn1—N3—C7	10.4 (3)	N3—C7—C8—C9	−178.6 (3)
N1—Zn1—N3—Se2	105.33 (13)	C12—C7—C8—C9	0.4 (4)
Cl2—Zn1—N3—Se2	−12.50 (13)	C7—C8—C9—C10	2.1 (4)
Cl1—Zn1—N3—Se2	−143.86 (10)	C8—C9—C10—C11	−2.1 (5)
N3—Se2—N4—C12	0.16 (19)	C9—C10—C11—C12	−0.6 (4)
Se1—N1—C1—C2	−178.1 (2)	Se2—N4—C12—C11	−177.8 (2)
Zn1—N1—C1—C2	18.7 (4)	Se2—N4—C12—C7	1.5 (3)
Se1—N1—C1—C6	1.8 (3)	C10—C11—C12—N4	−177.9 (3)
Zn1—N1—C1—C6	−161.37 (18)	C10—C11—C12—C7	2.9 (4)
N1—C1—C2—C3	−179.3 (3)	N3—C7—C12—N4	−3.1 (3)
C6—C1—C2—C3	0.8 (4)	C8—C7—C12—N4	177.8 (2)
C1—C2—C3—C4	0.8 (5)	N3—C7—C12—C11	176.2 (2)
C2—C3—C4—C5	−1.3 (5)	C8—C7—C12—C11	−2.9 (4)

Symmetry codes: (i) *x*, *y*−1, *z*; (ii) *x*, *y*+1, *z*.
